# Muscular cystic hydatidosis: case report

**DOI:** 10.1186/1471-2334-7-23

**Published:** 2007-03-30

**Authors:** Sonia Vicidomini, Gabriella Cancrini, Simona Gabrielli, Riccardo Naspetti, Alessandro Bartoloni

**Affiliations:** 1 Malattie Infettive e Tropicali, Azienda Ospedaliero-Universitaria Careggi, Viale Morgagni 85, 50134 Florence, Italy; 2 Dipartimento di Scienze di Sanità Pubblica, Università "La Sapienza", P.le Aldo Moro 5, 00185 Rome, Italy; 3 Chirurgia Generale 3, Azienda Ospedaliero-Universitaria Careggi Viale Morgagni 85, 50134 Florence, Italy

## Abstract

**Background:**

Hydatidosis is a zoonosis caused by *Echinococcus granulosus*, and ingesting eggs released through the faeces from infected dogs infects humans. The location of the hydatid cysts is mostly hepatic and/or pulmonary, whereas musculoskeletal hydatidosis is very rare.

**Case presentation:**

We report an unusual case of primary muscular hydatidosis in proximity of the big adductor in a young Sicilian man. The patient, 34 years old, was admitted to the Department of Infectious and Tropical Diseases for ultrasonographic detection, with successive confirmation by magnetic resonance imaging, of an ovular mass (13 × 8 cm) in the big adductor of the left thigh, cyst-like, and containing several small cystic formations. Serological tests for hydatidosis gave negative results. A second drawing of blood was done 10 days after the first one and showed an increase in the antibody titer for hydatidosis. The patient was submitted to surgical excision of the lesion with perioperatory prophylaxis with albendazole. The histopathological examination of the bioptic material was not diriment in the diagnosis, therefore further tests were performed: additional serological tests for hydatidosis for the evaluation of IgE and IgG serotype (Western Blot and REAST), and molecular analysis of the excised material. These more specific serological tests gave positive results for hydatidosis, and the sequencing of the polymerase chain reaction products from the cyst evidenced *E. granulosus *DNA, genotype G1. Any post-surgery complications was observed during 6 following months.

**Conclusion:**

Cystic hydatidosis should always be considered in the differential diagnosis of any cystic mass, regardless of its location, also in epidemiological contests less suggestive of the disease. The diagnosis should be achieved by taking into consideration the clinical aspects, the epidemiology of the disease, the imaging and immunological tests but, as demonstrated in this case, without neglecting the numerous possibilities offered by new serological devices and modern day molecular biology techniques.

## Background

Hydatidosis is a zoonotic infection caused by tapeworms belonging to the class Cestoda, in the family Taeniidae, of the genus *Echinococcus*. The *Echinococcus granulosus *species, which is responsible for cystic hydatidosis, has an almost ubiquitous diffusion. South America, Central Asia and the Mediterranean basin [1] must be considered highly endemic areas. The incidence of the disease in Italy is rated high in Sardinia and Sicily, medium in the Central-South regions, and low in the remaining areas [2]. The adult worm (3 to 6 mm long) lives in the small intestine of the definitive hosts, i.e. dogs or other canids [1]. Gravid proglottids containing infective eggs are shed daily through the faeces. After ingestion by a suitable intermediate host (usually herbivores like sheep, goats, swine, cattle, horses, camels, but occasionally also humans), the eggs hatch in the small intestine releasing a hooked larva called oncosphere. It penetrates the intestinal wall by means of its six hooks and migrates through the circulatory system reaching various organs, mainly the liver and lungs. Here the oncosphere loses the hooks and develops into a cyst that enlarges gradually. Usually by the fifth month, the wall of the cyst differentiates into an outer laminated non-nucleated layer and an inner nucleated germinal layer. The inner layer produces protoscolices and daughter cysts that fill the cyst interior, and can be attached or floating free within the cyst fluid. The dog becomes infected after swallowing the cyst-containing organs of the slaughtered parasitized herbivores. The ingested protoscolices attach to the intestinal mucosa, and develop into adult stage tapeworms within 32–80 days. Humans are accidental hosts that become infected by ingesting the eggs and, just like the aforementioned herbivorous hosts, allow the development of cysts in various organs. The growth rate of the cysts is about 1 cm per year. The size of the cysts varies between 1 and 15 cm, even though descriptions of cysts of up to 20 cm in diameter can be found in literature [1]. Cysts are typically univesicular, but sometimes small daughter cysts similar to the mother cyst, can be found in their interior. Hydatids are mostly found in the liver and/or in lungs for physiopathological reasons, but several other encysting sites are possible, bone included. The musculoskeletal involvement has been registered in only 1–4% of the cases [3]. It has been hypothesized that the presence of lactic acid in the muscles does not allow the larvae to grow into cysts [4]. Nevertheless, some cases of primary muscular hydatidosis at various sites have been reported, i.e. thoracic wall [5], sartorius [6,7], biceps brachii [4], supraspinatus [8], gluteus [9], pterygoideus [10] and soleus muscles [11], whereas, only few cases of primary subcutaneous hydatidosis of the inferior limbs have been reported [12-15]. In this study we report a case of muscular cystic hydatidosis of the adductor magnus muscle in a young Italian man from Sicily. We would like to point out that, regardless of the site involved, this zoonotic infection should always be considered in the differential diagnosis of any cystic lesion. Furthermore, it is extremely important to use all the available diagnostic methodologies to reach a definitive diagnosis.

## Case presentation

**Figure 1:**
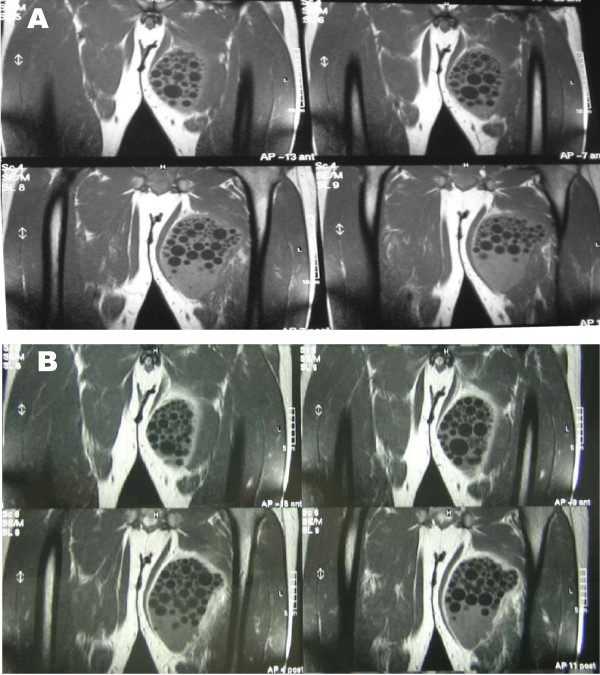
MRI of the left thigh, sequence T1-weighted: a) precontrast image: "ovular mass in the adductor magnus muscle, with a cystic aspect, dimensions 13 × 8 cm containing numerous cystic formations with regular outlines; b) after contrast image: "the lesion appears surrounded by a thin wall homogeneously impregnated after the injection of paramagnetic contrast medium while there is no contrastographic enhancement of the content of the lesion".

**Figure 2:**
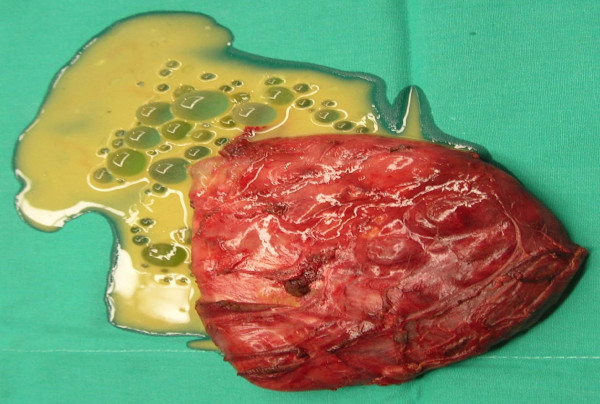
Macroscopic aspect of the hydatid cyst after the surgical excision.

The patient, C. L., a 34 year old male, was a bricklayer born in Sicily (Southern Italy), but had lived in the province of Florence (Tuscany region, Central Italy) for the past 10 years. He was admitted in our ward following the ultrasonographic detection of a cyst-like ovular mass (13 × 8 cm) which contained several small cystic formations. The cyst was in the adductor magnus muscle of the left thigh, and it was later confirmed by magnetic resonance imaging (MRI) (Figure 1). The patient had been complaining for approximately one month about a painful mass growing on his left thigh. Therefore, his physician prescribed imaging examinations, and eventually referred him to us. Our physical exam revealed a palpable mass with hard consistency, and no signs of erythema. The patient reported neither fever nor systemic symptomatology. The blood cell count was normal except for a relatively slight hypereosinophilia (white blood cells 6700/mmc, eosinophils 8.9%). Abdominal ultrasonography and chest X-ray were normal, and the serological tests for hydatidosis were negative (Echinococcosis IHA, Fumouze Diagnostics, Levallois Perret, France: 1:80, n.v. <1:160; Echinococcus granulosus IFA, Bios GmbH Munchen, Germany: negative). The same tests were repeated 10 days later and the results showed a weak increase in the antibody titre (IHA 1:320; IFA 1:40, n.v. <40). The patient was evaluated for, and then submitted to the surgical ablation of the lesion, while receiving chemoprophylaxis with albendazole 400 mg bid. Ten days after the surgical excision the patient stopped the perioperative prophylaxis. At a first glance, the excised material resembled a big cyst containing more than 60 translucent daughter cysts immersed in a clear liquid (Figure 2). The subsequent histopathological examination showed "an inflammatory tissue reaction surrounding a parasite-like cyst". Additional serological tests and bio-molecular diagnostics were then used to further examine the two available serum samples and the excised material respectively, with the aim to define the nature of the lesion. Enzyme immunoassay for specific IgG (EIA Echinococcus Ab, Cypress Diagnostics, Langdorp, Belgium) and reversed-enzyme-allergo-sorbent-test for specific IgE (REAST Allergyzen IgE, ZenTech, Angleur, Belgium) resulted positive only on the second blood sample. Whereas the Western Blot assay (WB), for specific *E. granulosus *proteins, corresponding to the band of 7 and 26–28 Kda (WB Echinococcus IgG, LDBIO Diagnostics, Lyon, France), resulted positive on both serum samples. As for the bio-molecular analysis, genomic DNA was extracted (Wizard SV Genomic DNA Purification kit, Promega, USA) from the cyst wall and from its liquid. A polymerase chain reaction (PCR) protocol was applied which amplified a 373 bp fragment of the mitochondrial 12S rRNA gene common to almost 12 Cestoda species, *E. granulosus *included [16]. Amplification products were gel-purified (Immolase DNA Purification Kit, Bioline, UK), and sequenced (MWG-Biotech AG, Germany). The sequences obtained were assembled using the program MEGA 3.1 [17]. A comparison with sequences available in the GenBank was made to identify genetic similarities with already known cestode sequences. Molecular diagnostics identified *E. granulosus *DNA, genotype G1 (98% homology). All five serological tests of the 8-week-after-surgery follow-up exam were negative. The patient underwent follow-up visits within the next 12 months without any occurrence of post-surgery complications.

## Conclusion

Hydatidosis is a serious public health problem in some areas of the World. Clandestine slaughtering together with climatic and ecological conditions, favour the spreading of the parasite among animals and contribute to maintain high infection rates in humans. Unfortunately, this practice is still very common in some areas of Italy despite the more severe regulations, which were adopted after the cases of human bovine spongiform encephalopathy (BSE). Early diagnosis of this zoonosis can be difficult, even in the more common hepatic and/or pulmonary cysts. The identification actually becomes more complex when the cysts are located in unusual sites, such as the musculoskeletal hydatidosis, and the parasitic aetiology is therefore often unsuspected. Hydatidosis should always be considered when a patient coming from an endemic area, shows a slow growing mass in the soft tissues. In our case, although we had diagnostic images highly suggestive of cystic hydatidosis, we did not have any other elements to support this hypothesis. As a matter of fact, even though our patient was originally from Sicily, an Italian region considered a high incidence area for hydatidosis; he had been living in Tuscany for the past 10 years, a middle incidence area for the disease. The anamnesis was negative for direct contact with dogs or stock farm environment. The patient had noticed a rapid (one month) increase in size of the swelling of the thigh. Imaging showed no evidence of lesions in the liver and lungs. Initial serologic screening tests, IHA and IFA, were negative. Surgical excision of the lesion was prompted by the need of reaching a definitive diagnosis, under perioperative chemoprophylaxis with albendazole. The patient had no post-surgery complications. Serological tests performed at the end of the perioperative prophylaxis and repeated two months later resulted negative. Microscopic and histopathologic findings showed the presence of some morphological features of a parasitic cyst, but it was not conclusive in confirming the presumptive diagnosis. Molecular testing of the surgical specimen allowed us to identify *E. granulosus *DNA, genotype G1, in the nucleated inner layer of the cyst wall. The experience here reported suggests that cystic hydatidosis should always be considered in the differential diagnosis of any cystic mass, regardless of its location, and of the epidemiological contexts which might be less suggestive of the disease. The diagnosis, as confirmed by this case, should be made by taking into account the clinical aspects and imaging analysis, but also the serological tests. IHA and IFA confirmed to be suboptimal tests, especially in extra-hepatic localizations, whereas WB which is known to be the most specific assay for *E. granulosus*, showed higher sensitivity. Furthermore, the additional use of molecular diagnostics may be a helpful tool to validate an indirect (presumptive) diagnosis. In this case, we identified a strain of *E. granulosus*, genotype G1, which is commonly found in sheep [18]. These biomolecular findings allowed us to establish that sheep farming environment or contaminated foods coming from the same area were our patient's source of infection. In addition, this diagnostic tool allowed us to exclude areas where cattle, swine, horses or goats were bred.

## Competing interests

The author(s) declare that they have no competing interests.

## Authors' contributions

SV participated to the clinical management and drafted the manuscript.

GC coordinated the parasitological analysis and helped to draft the manuscript.

SG carried out the molecular analysis.

NR participated to the clinical management and performed the surgical ablation of the lesion.

AB coordinated the clinical management and helped to draft the manuscript.

All authors read and approved the final manuscript.

## Pre-publication history

The pre-publication history for this paper can be accessed here:


